# Buccal DNA global methylation and cognitive performance in stunted children under five years of age

**DOI:** 10.7555/JBR.37.20230295

**Published:** 2024-08-16

**Authors:** Ahmad Rusdan Handoyo Utomo, Yusnita Yusnita, Siti Maulidya Sari, Octaviani Indrasari Ranakusuma, Sunu Bagaskara, Wening Sari, Yulia Suciati, Anggi Puspa Nur Hidayati, Silviatun Nihayah, Catur Anggono Putro, Neni Nurainy

**Affiliations:** 1 Graduate School of Biomedical Science, Universitas YARSI, Central Jakarta, DKI Jakarta 10510, Indonesia; 2 Faculty of Medicine, Universitas YARSI, Central Jakarta, DKI Jakarta 10510, Indonesia; 3 Faculty of Psychology, Universitas YARSI, Central Jakarta, DKI Jakarta 10510, Indonesia; 4 Development of Translational Biopharmaceutical Products Division, Bandung, West Java 40161, Indonesia

**Keywords:** global DNA methylation, 5mC, ELISA, stunting, cognitive

## Abstract

The prevalence of stunting in Indonesian children under five years of age is approximately 20%. Chronic maternal malnutrition contributes to the risk of stunting by affecting global DNA methylation. In the present study, we aimed to evaluate the levels of 5-methyl-cytosine (5mC) as a surrogate marker of global DNA methylation in buccal swabs and its potential association with the risk of stunting and cognitive performance. The levels of 5mC were measured using an enzyme-linked immunosorbent assay. The Wechsler Preschool and Primary Scale of Intelligence (WPPSI) was used to measure cognitive function. Buccal swab DNA samples and anthropometric data were collected from a total of 231 children aged zero to five years. In this cross-sectional cohort, the prevalence of stunting was 37% in 138 children aged zero to two years and 30% in 93 children aged over two years. The univariable analysis revealed that the levels of 5mC in buccal swab DNA were significantly lower in severely stunted children (median, 2.84; interquartile range [IQR], 2.39–4.62) and children aged less than two years (median, 2.81; IQR, 2.53–4.62) than those in normal children (median, 3.75; IQR, 2.80–4.74; *P*-value, 0.028) and children aged over four years (median, 4.01; IQR, 3.39–4.87; *P*-value < 0.001), respectively. We also found that the average cognitive scores tended to be low in boys and stunted children, although the differences were not statistically significant. Furthermore, the levels of 5mC found in buccal swab and mouthwash DNA were not associated with cognitive scores.

## Introduction

Stunting, defined by the World Health Organization as having a height-for-age *Z* score (HAZ) less than –2 standard deviations^[1]^, is a public health burden, because it reduces quality of life in adulthood and increases the risk of non-communicable diseases^[2]^ and low cognitive performance^[3]^. A national nutrition survey conducted by the Ministry of Health Directorate of the Republic of Indonesia in 2017 reported a stunting prevalence of approximately 20.1% among children under two years of age^[4]^. Regional clusters in Indonesia have a higher prevalence of stunting than the national average. For example, in the Pandeglang district in western Java, the prevalence of stunting is about 30%^[5]^.

Chronic malnutrition is an established risk factor for stunting, particularly maternal malnutrition during the first 1000 days following conception^[6]^. During this critical period, the totipotency of embryonic stem cells is essential for healthy development, which is achieved by erasing parental 5-methyl-cytosine (5mC) from the fetal genome^[7]^. 5mC is subsequently reintroduced into the fetal genome to promote tissue growth and differentiation. Therefore, the reintroduction of 5mC may be affected by inadequate maternal nutritional intake^[8]^. During embryonic development, the one-carbon metabolism pathway plays a crucial role in providing methyl groups (such as folate, betaine, methionine, and serine) and cofactors (*e.g.*, vitamins B2, B6, and B12) necessary for DNA methylation^[9]^. Maternal diet may affect DNA methylation in infants^[10]^. For example, a deficiency of methyl groups or cofactors in the maternal diet has been linked to DNA hypomethylation in Jamaican children^[11]^. Conversely, an epigenetic study in slum areas of Bangladesh among children aged two to three years demonstrated a significant association between lower protein and calorie intake and high global DNA methylation. However, whether malnutrition-induced stunting is associated with low or high global DNA methylation remains unclear and requires further study.

Stunting may lead to a poor neural development, affecting cognitive performance during school years and later in adulthood^[2]^. More importantly, persistent early-onset stunting leads to low cognitive scores^[3]^. Poor cognitive function may result from defects in neural development, as observed in animal models of malnutrition^[12]^.

Efforts to prevent stunting have faced many challenges, including low compliance among participants^[13–14]^, the lack of relevant biomarkers for specific micronutrient deficiencies^[15]^, and non-standardized anthropometric measurements^[16]^, which may affect the accuracy of stunting prevalence estimates. Recent studies have shown that buccal cells and neurons share similar global DNA methylation patterns^[17]^. Therefore, buccal cells, which are readily accessible in children, may serve as surrogate specimens for cognitive studies.

In the present study, we aimed to investigate the association between 5mC levels in buccal DNA, collected by a minimally invasive method, and stunting or cognitive performance.

## Subjects and methods

### Study population and design

**Figure 1:**
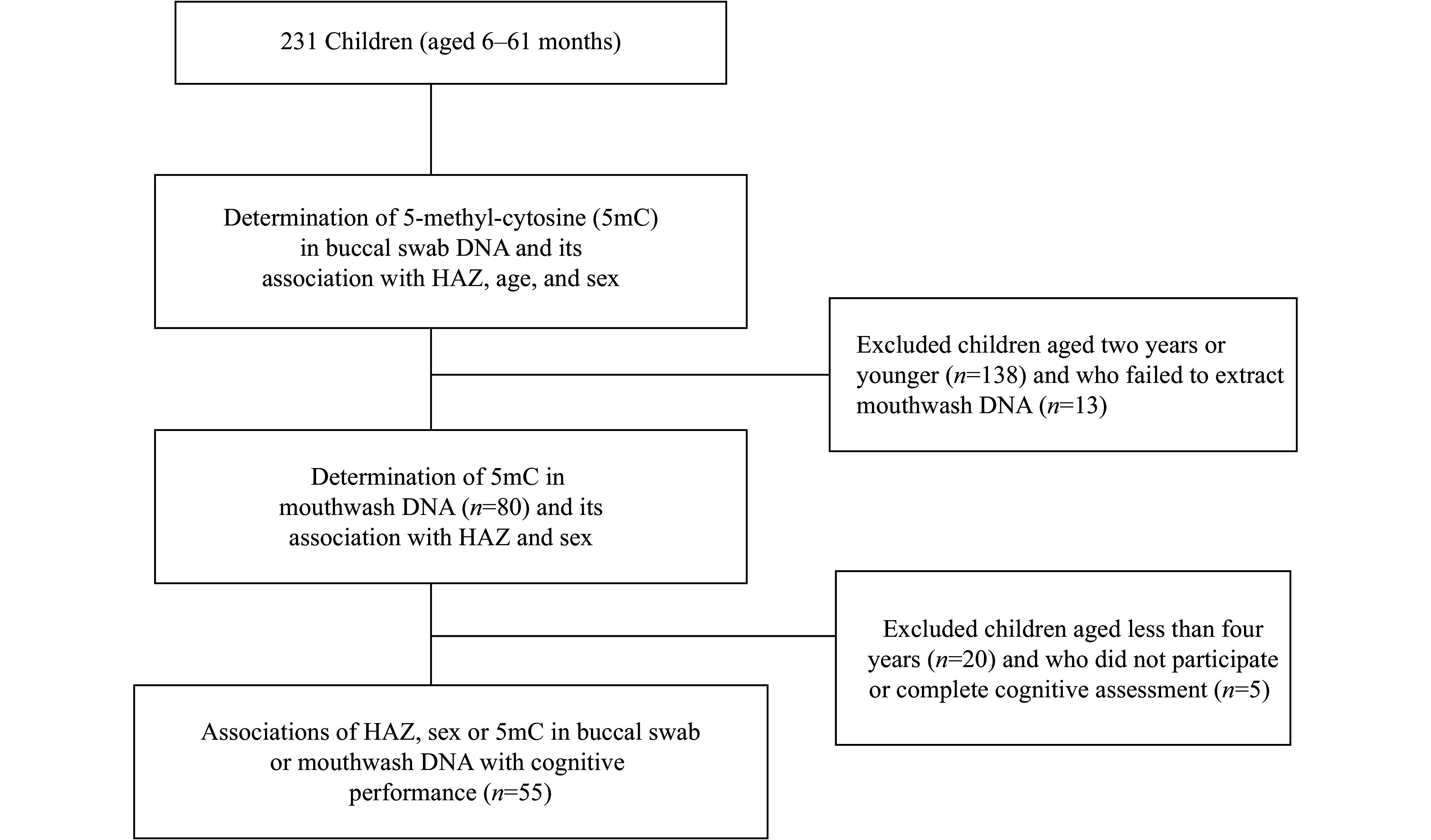
Diagram showing subject recruitment, inclusion criteria, and planned analyses. Abbreviation: HAZ, height-for-age *Z* score.

The present cross-sectional study enrolled 231 children (aged zero to five years) from local community health facilities in three villages (Kadumaneuh, Kadubelang, and Medong) in Pandeglang district, Banten province, Indonesia. The prevalence of stunting in Pandeglang, especially in Kadumaneuh, has been estimated at approximately 30% according to previous studies^[5,18]^. The sample size needed for the analysis was not calculated, because no references in the literature had described global DNA methylation in buccal specimens in children with stunting. However, cross-sectional studies using buccal DNA as a source of global DNA methylation in non-stunted children recruited between 48 and 73 children^[19–20]^. Considering the budget, we conducted a consecutive sampling of children who attended community healthcare facilities in the villages on September 10, 2022, from 9 a.m. to 12 p.m. to collect their buccal cells and anthropometric measurements. There were four healthcare facilities in each village, amounting to a total of 12 recruitment sites. Cognitive measurements were performed on children aged four years or older within one month after recruitment. A diagram of subject recruitment, inclusion criteria, and analyses is shown in ***Fig. 1***.

Buccal DNA samples were collected from all 231 children, consisting of 138 children aged two years or younger and 93 children older than two years. In addition, children older than two years were invited to participate in DNA mouthwash collection. Mouthwash DNA was successfully collected from 80 children. Since stunting may also affect cognitive function, a subset of these 80 children, *i.e.*, children aged four years or older (*n* = 55), participated in and completed the cognitive evaluation. After a detailed explanation of the study, we obtained the informed consent from their parents or guardians, and the present study was conducted following the Helsinki Declaration. This study was approved by the YARSI Ethics Committee No. 238/KEP-LPUY/VIII/2022.

### Anthropometric status

The height/length of the children was measured directly through physical examination, which was recorded in centimeters according to the standards of the Ministry of Health of the Republic of Indonesia, as previously described^[5]^. Briefly, the measurement of body length for children under two years old was conducted in a supine position using the GEA WB-C Stature Gauge (Changzhou Wujin Weighing Apparatus Factory, Changzhou, China). For children over two years old, height was measured in a standing position using the GEA SH2A Stature Meter (Changzhou Wujin Weighing Apparatus Factory). Length and height measurements were recorded to the nearest 0.1 cm. All measurements were performed by trained medical students with expertise in anthropometric assessment. The height-for-age *Z* score (HAZ) for children was calculated using the WHO growth standard. Children with −2 ≤ HAZ ≤ 2 were considered normal, −3 ≤ HAZ < −2 were considered stunted, and HAZ < −3 were considered severely stunted.

### DNA collection and extraction

Buccal swab DNA was collected from all participants using commercial buccal DNA isolation kits (BioSaliva Kit, Biofarma, Bandung, Indonesia). Parents were asked not to give meals to their children 30 min before sample collection. To collect the buccal cells, the trained medical students rubbed a sterile swab head against the inside cheeks of the child for 20 rounds on each cheek. In addition, children aged four to five years were also asked to take about 2.5 mL of mouthwash liquid provided by the kit solution and spit it into the preservative solution^[21]^. The samples were stored in a sterile tube at room temperature and transported to the YARSI University molecular testing laboratory. DNA was extracted using an automated DNA extraction kit (Maxwell^®^ RSC Blood DNA Kit, Cat. AS1400, Promega, Madison, WI, USA) with magnetic bead technology (Maxwell^®^ RSC Instrument, Promega). The DNA concentration was quantified using spectrophotometry (Tecan Infinite M200 Pro, Switzerland).

### Epigenetic measurement of global DNA methylation

Global methylation of child buccal swab DNA samples was analyzed using an indirect ELISA-based commercial kit (5-mC DNA ELISA Kit; Cat. D5325; Zymo Research, Irvine, CA, USA), as previously described. Briefly, 100 ng of input DNA was bound to the wells, and methylated DNA was detected using antibodies to 5mC and then quantified colorimetrically by reading absorbance at 405 nm using an ELISA plate reader (Tecan Infinite M200 Pro, Switzerland). The standard curve was generated using the absorbance values of seven standards made by mixing positive and negative controls provided by the kit. The final standard methylation concentrations were 0%, 5%, 10%, 25%, 50%, 75%, and 100%, respectively. The 5mC levels for unknown DNA samples were calculated as follows: %5mC = e^(Absorbance−Intercept)/Slope^.

### Cognitive evaluation

Child cognitive function was evaluated using the Wechsler Preschool and Primary Scale of Intelligence (WPPSI), which is suitable for children aged four to 7.3 years, as described previously^[22]^. The trained psychologist assistants conducted the assessment, which was closely supervised by a licensed psychologist. The assessment was administered at each child's home to minimize unfamiliar surroundings that may distract children's attention during the test. The administration of the test took 60–70 min. The test was paused when the child looked tired or bored. During the break, the assistant offered the child a snack or drink and talked about a toy or game the child favored. The break usually lasted five minutes before the assistant asked the participant to continue the test. When the child refused to continue, the assistant stopped the assessment and returned the next day.

Cognitive function was measured using the Indonesian version of the WPPSI published by the Faculty of Psychology, Universitas Indonesia^[22]^. The WPPSI consists of two scales: the Verbal Scale and the Performance Scale. The Verbal Scale was measured by five subtests: information, vocabulary, comprehension, similarities, and arithmetic. The Performance Scale was measured by another five subtests: animal house, picture completion, mazes, geometric design, and block design. The raw score of each subtest was standardized according to age group before summing the scores for each scale and converting them into verbal intelligence quotient (VIQ) and performance intelligence quotient (PIQ). The standard scores of the Verbal and Performance Scales were summed and converted into full-scale intelligence quotient (FIQ)^[22]^.

### Statistical analysis

We used the Chi-square test to compare the proportions of normal, stunted, and severely stunted children in different independent variables, such as sex, age, and villages. Because the values of 5mC levels were not normally distributed, we compared the median of 5mC levels in multiple groups of HAZ status (*i.e.*, normal, stunted, and severely stunted children) and age (zero to 24 months, 24 to 48 months, and older than 48 months) using the Kruskal-Wallis rank test. When the Kruskal-Wallis test showed statistical significance, post hoc Dunn-Bonferroni tests were calculated to reveal differences among different subgroups (HAZ status and age groups). The Mann-Whitney test was used to compare the median values of 5mC levels between boys and girls. Binary logistic regression was used to test numerical data (5mC levels) as a predictor of binary stunting outcomes in different age groups. To determine the relationship between global DNA methylation and cognitive function, we used a linear regression model as described previously. A two-sided *P*-value < 0.05 was considered statistically significant. All statistical analyses were performed using StatPlus for Mac statistical software (AnalystSoft Inc., Walnut, CA, USA), GraphPad Prism version 10.0.0 for Windows (GraphPad Software, Boston, MA, USA), and DATAtab: Online Statistics Calculator (DATAtab e.U. Graz, Austria).

## Results

### Demographics of participants

Children ranged in age from two to 73 months, with a mean age of 28.4 months (SD = 20.1 months) and a median age of 19 months (IQR, 12–49 months). As shown in ***Table 1***, the prevalence of stunting (−3 ≤ HAZ < −2) and severe stunting (HAZ < −3) in this cohort was 21% and 13% out of 231 subjects, respectively. Moreover, the prevalence of stunting was 37% in children aged zero to two years and 30% in children older than two years. Approximately 57% of the subjects were male, and 60% were aged zero to two years. Of the three villages, Kadumaneuh had the highest prevalence of severe stunting. The average concentration of buccal DNA and the corresponding 5mC levels were 28.3 ng/μL and 4.06%, respectively.

**Table 1:** Demographics of 231 children aged zero to five years who participated in the present study

Variables	*n* (%)
Age (months)	
0–24	138 (60)
24–48	14 (6)
≥48	79 (34)
Sex	
Male	131 (57)
Female	100 (43)
Outcomes	
Normal (−2≤HAZ≤ 2)	152 (66)
Stunting (−3≤HAZ<−2)	49 (21)
Severe stunting (HAZ<−3)	30 (13)
Villages (normal, stunting, and severe stunting)	
Kadubelang (*N*=73)	47 (65), 17 (23), 9 (12)
Kadumaneuh (*N*=72)	43 (60), 14 (19), 15 (21)
Medong (*N*=86)	61 (71), 19 (22), 6 (7)
Abbreviation: HAZ, height-for-age *Z* score.	

### Stunting prevalence among participants

As shown in ***Table 2***, stunting outcomes were not significantly associated with age, sex, or the villages in which the children lived (*P*-values > 0.05).

**Table 2:** Proportion of the stunting outcome according to independent variables

Variables	Normal [*n* (%)]	Stunting [*n* (%)]	Severe stunting [*n* (%)]	*P*-value
Age (months)				
0–24	87 (57)	27 (55)	24 (80)	0.204
24–48	9 (6)	4 (8)	1 (33)	
≥48	56 (37)	18 (37)	5 (17)	
Sex				
Male	90 (59)	29 (59)	12 (40)	0.140
Female	62 (41)	20 (41)	18 (60)	
Villages				
Kadubelang	47 (31)	16 (32)	9 (30)	0.776
Kadumaneuh	44 (29)	14 (29)	15 (50)	
Medong	61 (40)	19 (39)	6 (20)	
The height-for-age *Z* score (HAZ) for children was calculated using the WHO growth standard. Children with −2 ≤ HAZ ≤ 2 were considered normal, −3 ≤ HAZ < −2 were considered stunted, and HAZ < −3 were considered severely stunted. *P*-value was calculated by the Chi-square analysis.				

### Association of the 5mC levels in buccal swab DNA with age, sex, and HAZ

As shown in ***Table 3***, the levels of 5mC were significantly associated with age, sex, and HAZ status. Specifically, normal children had significantly higher 5mC levels than severely stunted children, and the youngest age group (zero to 24 months) had the lowest levels of 5mC, compared with older age groups.

**Table 3:** Association of 5-methyl-cytosine levels in buccal swab DNA with age, sex, and stunting

Variables	5mC levels in % [median (IQR)]	*P*
Age (months)		**<0.001** ^a^
0–24	2.81 (2.53–4.62)	
24–48	4.09 (3.76–4.56)	
>48	4.01 (3.39–4.87)	
Post hoc analysis		
0–24 months *vs.* 24–48 months		**0.038** ^b^
0–24 months *vs.* >48 months		**< 0.001** ^b^
24–48 months *vs.* >48 months		1.000^b^
Sex		**0.0187** ^**c**^
Male	3.83 (2.79–4.83)	
Female	3.25 (2.60–4.48)	
Outcomes		**0.031** ^**a**^
Normal (−2≤HAZ≤2)	3.75 (2.80–4.74)	
Stunting (−3≤HAZ<−2)	3.64 (2.70–4.57)	
Severe stunting (HAZ<−3)	2.84 (2.39–4.62)	
Post hoc analysis		
Normal *vs.* stunting		1.000^b^
Normal *vs.* severe stunting		**0.028** ^b^
Stunting *vs.* severe stunting		0.132^b^
^a^The Kruskal-Wallis test was employed to compare the median of multiple groups.^b^Dunn-Bonferroni was employed as a post hoc analysis.^c^The Mann-Whitney test was employed to compare the median of the two groups.Bold font indicates *P*-values < 0.05.Abbreviations: HAZ, height-for-age *Z* score; IQR, interquartile range.		

**Figure 2:**
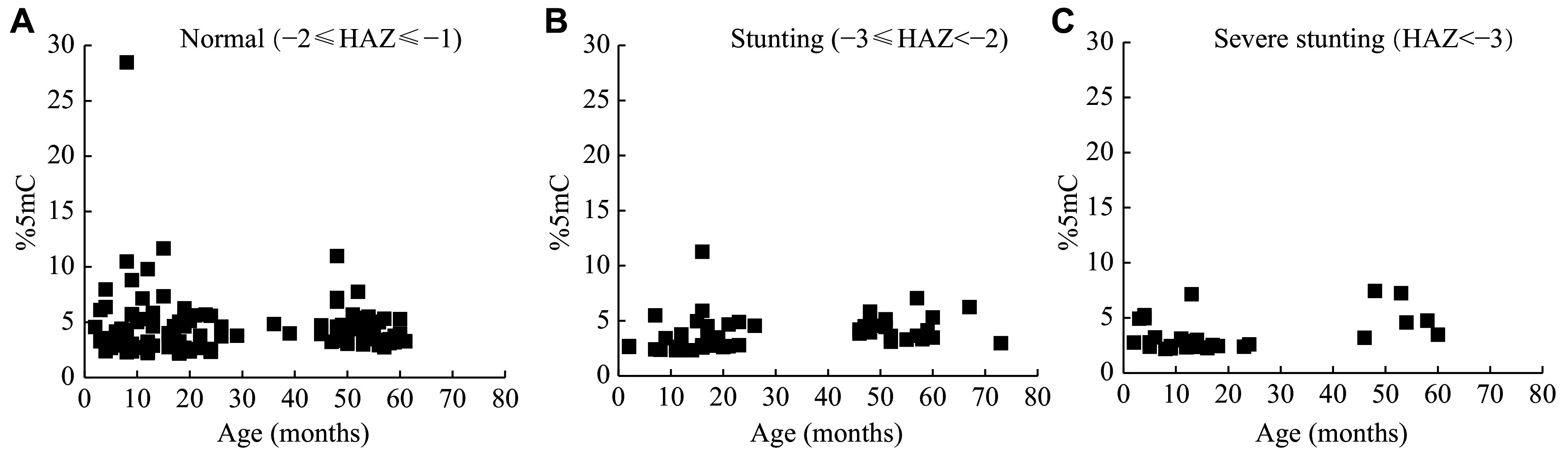
Distribution of 5mC levels in buccal swab DNA in different age groups and HAZ status. Scatter plots were used to visualize the relationships between 5mC levels and age. Each dot represents a single subject. The left panel represents children with normal HAZ (−2 ≤ HAZ ≤ 2), the middle panel stunting (−3 ≤ HAZ < −2), and the right panel severe stunting (HAZ < −3). Abbreviations: HAZ, height-for-age *Z* score; %5mC, percentage of 5-methyl-cytosine.

The distribution of 5mC levels by age and HAZ status is shown in ***Fig. 2***. Because 5mC levels were not normally distributed, the Spearman correlation analysis was used to examine the correlations between 5mC levels and age in different HAZ statuses. As shown in ***Table 4***, a significant correlation was found between age and 5mC levels in stunted children (−3 ≤ HAZ < −2), but not in normal (−2 ≤ HAZ ≤ 2) or severely stunted (HAZ < −3) children.

**Table 4:** Correlation of age and 5mC levels in different HAZ status

Outcomes	*n*	*r*	95% CI	*P*
Normal (−2≤HAZ≤2)	152	0.1345	−0.02998–0.2919	0.0985
Stunting (−3≤HAZ<−2)	49	0.4439	0.1776–0.6496	**0.0014**
Severe stunting (HAZ<−3)	30	0.1363	−0.2460–0.4819	0.1363
Spearman correlation analysis was performed. Bold font indicates *P*-values < 0.05. Abbreviations: HAZ, height-for-age *Z* score; CI, confidence interval.				

Although 5mC levels were lower in severely stunted children, we tested whether this association remained after adjusting for age and sex in this cohort (*n* = 231). The results of multivariate logistic regression analysis showed that the outcome of stunting (including both stunted and severely stunted children with an HAZ < −2) was not significantly associated with age (coefficient, 0.004; 95% confidence interval [CI], −0.010–0.018), 5mC levels (coefficient, 0.099; 95% CI, −0.039–0.278), and sex (coefficient, 0.252; 95% CI, −0.304–0.808).

### Association of 5mC levels in mouthwash DNA with HAZ and sex

Mouthwash DNA was another source of non-invasive DNA collection, which did not require swabbing tools. Out of 93 children aged more than two years, mouthwash DNA was successfully extracted from 80 children. Therefore, there were 80 children with paired DNA collected from buccal swabs and mouthwash. The 5mC levels in swab DNA (median, 4.01; IQR, 3.40–4.70) were significantly higher than those in mouthwash DNA (median, 3.69; IQR, 3.27–4.20) (*P* = 0.018 by Wilcoxon matched-pairs signed rank test). There was no significant difference in 5mC levels in mouthwash DNA among children of different sexes and HAZ statuses (***Table 5***).

**Table 5:** Association of 5mC levels in mouthwash DNA with sex and HAZ in children older than two years

Variables	*n*	5mC levels [median (IQR)]	*P*
Sex			0.59^a^
Male	47	3.69 (3.26–4.03)	
Female	33	3.68 (3.38–4.23)	
HAZ			0.63^b^
−2≤HAZ≤2	51	3.68 (3.25–4.27)	
−3≤HAZ<−2	18	3.65 (3.39–4.09)	
HAZ<−3	11	3.96 (3.34–4.20)	
^a^Mann-Whitney was employed to compare median values of 5mC levels in males and females.^b^Kruskal-Wallis was employed to compare median values of 5mC levels in three different HAZ statuses.Abbreviations: HAZ, height-for-age *Z* score; IQR, interquartile range.			

### Association of 5mC levels, stunting, and cognitive performance

Of the 80 children, 55 aged four years and over completed the cognitive assessment. For the three cognitive scores (*i.e.*, PIQ, VIQ, and FIQ), the differences in performance attributed to sex and HAZ were not significant (***Table 6***). Of note, children who lived in Kadumaneuh village had significantly lower VIQ and FIQ scores than those living in the other two villages.

**Table 6:** Cognitive function according to sex, HAZ, and villages

Variables	*n*	PIQ [median (IQR)]	*P*	VIQ [median (IQR)]		FIQ [median (IQR)]	
Sex			0.983^a^		0.936^a^		0.996^a^
Male	32	99.5 (86–111)		100 (95–116)		101 (94–110)	
Female	23	100 (92–108)		104 (89–116)		101 (90–113)	
HAZ			0.797^a^		0.573^a^		0.825^a^
−2≤HAZ≤2	38	100 (85–108)		101 (94–117)		101 (94–111)	
HAZ<−2	17	99 (92–113)		97 (89–115)		99 (91–112)	
Villages			0.293^b^		0.0065^b^		0.019^b^
Medong	22	102 (97–111)		109 (99–121)		111 (97–115)	
Kadumaneuh	18	97 (80–105)		93 (85–100)		96 (79–103)	
Kadubelang	15	100 (92–108)		105 (98–113)		102 (99–106)	
^a^*P*-value by the Mann-Whitney test.^b^*P*-value by the Kruskal-Wallis ANOVA.Abbreviations: HAZ, height-for-age *Z* score; PIQ, performance intelligence quotient; VIQ, verbal intelligence quotient; FIQ, full-scale intelligence quotient; IQR, interquartile range.							

To assess different variables that might influence performance IQ scores, we performed multivariable regression analysis, and the results are shown in ***Table 7***. None of the variables had a significant association with cognitive performance.

**Table 7:** Association of 5mC levels in buccal swab and mouthwash DNA as well as other variables with cognition

Variables	PIQ				VIQ				FIQ		
	Coefficient (95% CI)	*P*	*R* ^*2*^		Coefficient (95% CI)	*P*	*R* ^*2*^		Coefficient (95% CI)	*P*	*R* ^*2*^
Sex (Female)	−0.0625 (−9.14–9.01)	0.98	0.10		3.05 (−6.20–12.31)	0.50	0.10		2.18 (−6.64–11.01)	0.62	0.10
Villages (Kadumaneuh)	−6.96 (−18.16–4.23)	0.21	0.36		−11.39 (−22.81–0.03)	0.050	0.36		−9.81 (−20.71–1.08)	0.07	0.36
Stunting outcomes (stunted)	−6.88 (−16.92–3.15)	0.17	0.16		−6.21 (−16.46–4.02)	0.23	0.16		−7.11 (−16.88–2.64)	0.14	0.16
5mC levels											
Buccal swab DNA^a^	1.33 (−2.02–4.68)	0.42	0.24		−0.21 (−3.63–3.21)	0.90	0.24		0.67 (−2.59–3.93)	0.68	0.24
Mouthwash DNA^b^	9.34 (−14.45–33.13)	0.43	0.07		7.18 (−17.08–31.46)	0.55	0.075		9.47 (−13.66–32.61)	0.41	0.07
^a^Values of 5mC levels in buccal swab DNA were left untransformed.^b^Values of 5mC levels in mouthwash DNA were natural logarithm transformed to obtain a normal distribution.Abbreviations: PIQ, performance intelligence quotient; VIQ, verbal intelligence quotient; FIQ, full-scale intelligence quotient; CI, confidence interval.											

## Discussion

In the present study, we successfully collected a sufficiently high concentration of DNA from buccal swabs and mouthwash for suitable epigenetic analysis. Both methods were non-invasive for children. We demonstrated that 5mC levels were higher in boys and older children than in girls and younger children, consistent with previous studies^[23]^. The exact mechanism underlying sex-specific global DNA methylation remains unclear but may involve the epigenetic regulation of steroid hormones^[24]^.

Iqbal *et al*^[25]^ investigated global DNA methylation in children aged two to three years using blood DNA samples and the ELISA method, reporting slightly higher 5mC levels in stunted children than in normal children, albeit the difference was not statistically significant. In the present study, where most children were under two years old, the 5mC levels in buccal DNA were significantly lower in severely stunted children than in normal children. This discrepancy may be influenced by the natural increase in global methylation during early childhood^[26]^.

Although the present study does not support the use of 5mC levels as a predictor of stunting, previous research demonstrates that the rapid increase in genome methylation during early normal childhood^[26]^ may be impaired in children with growth problems^[27]^. Therefore, global methylation levels may vary with age^[26]^. Older children with stunting may also exhibit higher levels of methylated DNA, because of increased susceptibility to bacterial infections and poor oral health, such as caries^[28]^. Bacterial DNA also contains 5mC, which complicates analysis using tools like ELISA as these methods cannot distinguish between bacterial and human DNA. It is estimated that 0.8% of all cytosines in *E*. *coli* DNA are methylated, compared with about 4% of all cytosine nucleotides in the human genome^[29]^.

In the present study, we showed that the 5mC levels in buccal swab DNA were higher in boys, consistent with findings from other studies using peripheral blood DNA^[25]^ and buccal swab DNA. However, this pattern was not observed in mouthwash DNA. The discrepancy between buccal swab and mouthwash DNA may be due to bacterial DNA methylation, which the ELISA method cannot distinguish from human DNA^[30]^.

Stunting is a public health concern because of its potential impact on cognitive function, with long-term consequences in adulthood^[31–32]^. However, in the present study, the differences in cognitive function between normal and stunted children were not statistically significant. Notably, children living in the village of Kadumaneuh had the lowest cognition scores, although multivariable regression analysis did not confirm this difference as statistically significant. Furthermore, mean scores (> 90) of PIQ, VIQ, and FIQ in stunted children were still considered normal within the general population. These findings do not support previous studies showing that stunted children have lower IQ^[33–34]^. Koshy *et al*^[35]^ also found that stunted children had significantly lower VIQ than non-stunted children at two, five, and nine years of age. However, these studies did not use the same measures. Aurora *et al*^[34]^ used the Colored Progressive Matrices (CPM), while Koshy *et al*^[35]^ used the Malin's Intelligence Scale of Indian Children (MISIC), an adaptation of the Wechsler Intelligence Scale for Children (WISC), both assessing children aged nine years. Venables *et al*^[33]^ examined the intelligence of three-year-old children using the WPPSI subtest and found direct correlations with VIQ and PIQ. Meanwhile, Mohd Nasir *et al*^[36]^ used the CPM to measure general cognitive function in children aged four to six years and found that height-for-age contributed to cognitive performance only after controlling for sociodemographic background and parental nutrition knowledge.

A meta-analysis showed that cognitive ability was highly dependent on social education and a stimulating environment^[37]^. Although nutritional supplements are important, the risk of low cognitive function may be minimized by providing children with social support. However, a prospective study would be ideal to follow cognitive performance in school at a later age in this cohort and assess the association with global DNA methylation or specific genes affected by methylation using the NextGen sequencing.

Using the linear regression model, one study showed a decreasing tendency (though insignificant) of all cognition scores for each increasing unit of 5mC levels in children aged four years^[38]^. Interestingly, we did not observe this decreasing trend for any of the cognitive parameters. The cause of this discrepancy is currently being investigated, including factors such as the small size of our cohort, the specific cognitive measurement tools used, and the exposure of our cohort to a stimulating environment.

A limitation of the present study was that we did not assess dietary intake in our cohort. Additionally, we did not analyze breastfeeding history, which may influence DNA methylation^[39]^. Stunting primarily results from chronic malnutrition during the first 1000 days post-gestation. Therefore, low levels of DNA methylation (or global hypomethylation) in a stunted cohort under the age of two years may reflect inadequate intakes of nutrients containing sufficient methyl donors or cofactors^[23]^. Since nutritional intervention is critical in the first 1000 days of child development, obtaining a methylation profile using a non-invasive method may help identify children under two years old who require targeted nutritional intervention.

Regarding cognitive assessment in stunted children, challenges arise when assessing preschool children, whose attention spans are often limited. Although test administrators were trained to build rapport and carefully administer the tasks, some obstacles arose during the assessment. The high dropout rate of the participants reflected these challenges, with children either refusing to continue the test on the following day or the caregivers taking them to family events, preventing completion of the second day of assessment. Despite these challenges, the assessment of cognitive function is important for the early identification of cognitive delays so that appropriate interventions may be implemented at an earlier stage.

In conclusion, we demonstrated that both buccal and mouthwash samples were suitable materials for a follow-up study to clarify the precise molecular mechanisms of epigenetic alterations affecting cognitive function in stunted children. These stunted children in this cohort may be followed up for further cognitive studies when they reach primary school age.
